# The Role of Leptin on the Organization and Expression of Cytoskeleton Elements in Nucleus Pulposus Cells

**DOI:** 10.1002/jor.22308

**Published:** 2013-01-17

**Authors:** Zheng Li, Jianxiong Shen, William Ka Kei Wu, Xin Yu, Jinqian Liang, Guixing Qiu, Jiaming Liu

**Affiliations:** 1 Department of Orthopedic Surgery, Peking Union Medical College Hospital, Peking Union Medical CollegeBeijing, China; 2 Department of Medicine and Therapeutics, Institute of Digestive Diseases, LKS Institute of Health Science, The Chinese University of Hong KongHong Kong, China

**Keywords:** leptin, cytoskeleton reorganization, nucleus pulposus cells, intervertebral disc degeneration

## Abstract

Obesity is an important risk factor for intervertebral disc degeneration and leptin is a biomarker of obesity. However, the expression of leptin receptors has not been determined in disc tissue. It is not known whether leptin has a direct effect on the nucleus pulposus (NP) cells. To determine whether the NP tissues and cells express leptin receptors (OBRa and OBRb) and whether leptin affects the organization and the expression of major cytoskeletal elements in NP cells. Messenger RNA (mRNA) and protein levels of OBRa and OBRb were measured by real-time PCR and Western blot, respectively, in NP tissues and cells. Immunofluorescence and real-time PCR and Western blot were performed to investigate the effect of leptin on cytoskeleton reorganization and expression. Results show that mRNA and proteins of OBRa and OBRb were expressed in all NP tissues and cells, and that OBRb expression was correlated with patients' body weight. Increased expression of β-actin and reorganization of F-actin were evident in leptin-stimulated NP cells. Leptin also induced vimentin expression but had no effect on β-tubulin in NP cells. These findings provide novel evidence supporting the possible involvement of leptin in the pathogenesis of intervertebral disc degeneration. © 2013 Orthopaedic Research Society. Published by Wiley Periodicals, Inc. J Orthop Res 31: 847–857, 2013

Low back pain is a common disorder, which has a lifetime prevalence of about 70%.^1^ Disc degeneration of the lumbar spine is considered to be one of the underlying factors of low back pain. Approximately 5.7 million people in the 45–64 age group are diagnosed with intervertebral disc degeneration each year in the United States.^2^ Nevertheless, the exact etiology of the disc degeneration is still unknown. Epidemiological studies suggest that lifestyle factors such as body weight, cigarette smoking, and exposure to vehicular vibration are implicated in disc degeneration.^3^ Obesity is a modifying factor and is associated with disc degeneration.^4^ Recent findings indicate that adiposity rather than simply excess in body mass is detrimental to the bone strength.^5^ There are growing interests in the field to study the contribution of metabolic factors associated with obesity in the pathogenesis of disc degeneration. However, the underlying molecular mechanisms accounting for the association between obesity and lumbar disc degeneration remain to be fully elucidated.

Leptin, a major adipocyte-derived adipocytokine, is a known biomarker of obesity. There is a tight association between circulating levels of leptin and both the body mass index (BMI) and the total amount of body fat. Leptin serum levels are about five times higher in obese people than in normal individuals, with an average of 40 and 8 ng/ml, respectively.^6^ Leptin not only controls appetite and body mass, but also takes part in the regulation of endocrine functions, metabolism, reproduction, immunity, cardiovascular pathophysiology, respiratory function, and wound healing, as well as osteoarthritis.^7^ Leptin acts via its receptors (OBR), which is encoded by the db gene. Several leptin receptor isoforms exists, including OBRa, OBRb, OB-Rc, OB-Rd, OB-Re, and OB-Rf, all of which share an extracellular domain of over 800 amino acids and a transmembrane domain of 34 amino acids and have a variable intracellular domain characteristic for each of the isoform. OBR can be classified into three classes: short, long, and secreted. However, only the long full-length isoform, OBRb, is considered to be the functional receptor. The almost universal distribution of OBRa and OBRb reflects the pleiotropic biological effects of leptin in non-adipose tissues, providing evidence for the extreme functional diversity of leptin.^8^ Previous studies have demonstrated the human intervertebral disc cells can secret detectable levels of leptin.^9^ Zhao et al.^10^ have also shown that disc cells express leptin functional receptor whose expression is correlated with the age of the patients. However, the role of leptin receptor in the pathogenesis of disc degeneration is not yet clear. Increase in body weight is known to upregulate OBR mRNA expression in adipose. Nevertheless, the contribution of body weight to the regulation of the leptin-receptor expression in human intervertebral disc remains elusive.

The intervertebral disc (IVD) is a heterogeneous structure that contributes to load support and flexibility in the spine. IVD is comprised of NP (NP) cells and annulus fibrosus (AF).^11^ The cytoskeleton of NP cells is characterized by a three-dimensional network consisting of actin microfilaments, tubulin microtubules, and vimentin intermediate filaments. The cytoskeleton is fundamental to the dynamic functions of the cells and plays important roles in cell division, motility, and protein trafficking and secretion. The behavior of mature intervertebral disc cells depends on the organization and composition of their cytoskeleton.^12^ Our recent work observed that leptin may be involved in the pathophysiology of osteoarthritis via induction of cytoskeletal reorganization in chondrocytes.^13^

Here, we hypothesized that OBR expressed in NP cells may play a functional role in obesity-related IVD degeneration. The aims of the present study is therefore to determine whether the NP tissues and NP cells express OBRa and OBRb and whether leptin affects the organization and the expression of major cytoskeletal elements in NP cells. Finding of this study will provide insights into the effect of obesity on the biochemical and morphological properties of NP cells in healthy and diseased tissue.

## MATERIALS AND METHODS

### Ethics Statement

Intervertebral disc tissue was obtained with fully informed written consent and the Clinical Research Ethics Committee of the Peking Union Medical College Hospital approval from patients undergoing discectomy.

### Patients and Samples

Human lumbar IVD samples from 45 cases in this study were obtained during surgical discectomy for radiculopathy with or without low back pain, aged 26–56 (mean, 48.0), among whom 26 were males and 19 were females. Patients who had spinal stenosis, spondylolisthesis, far lateral disc herniation, multiple disc herniations, or recurrent disc herniation were excluded. The duration of symptoms from onset to surgery ranged from 1 to 120 months (mean, 31.7 months). Routine MRI scans of the lumbar spine were taken for these patients before the operation; the degree of disc degeneration was graded from T2-weighted images using a modified Pfirrmann^14^ classification. According to the modified classification system of the International Society for the Study of the Lumbar Spine,^10^ 13 samples were protrusions, 12 were sequestration, 10 were subligamentous extrusion and 10 were transligamentous extrusion. Six of the 45 samples were obtained from the level of L3 to L4, 23 from L4 to L5, 16 from L5 to S1. Herniation tissues and granulation tissues were excluded. Tissues specimens were first washed thrice with phosphate-buffered saline (PBS) NP was separated from the AF using a stereotaxic microscope and then frozen in the liquid nitrogen.

### Isolation and Culture of Human NP Cells

The human NP cells were dissected from patient disc surgical specimens (eight donors, 28–40 years, and average Thompson degeneration grade 2–3). All of these NP tissues was isolated from patients underwent surgeries for disc degeneration (L4–L5) and not disc herniation, and therefore, contact between these tissues and cells outside of the disc, these are macrophages, endothelial cells and other immune cells, were minimal or nonexistent. No granulation tissue was present. NP cells were isolated and cultured as previously described.^15^ After isolation, NP cells were resuspended in DMEM containing 10% FBS (GIBCO, New York, NY), 100 µg/ml streptomycin, 100 U/ml penicillin and 1% l-glutamine, and then incubated at 37°C in a humidified atmosphere with 95% air and 5% CO_2_. The confluent cells were detached by trypsinization, seeded into 35 mm tissue culture dishes in complete culture medium (DMEM supplemented with 10% FBS, 100 µg/ml streptomycin, and 100 U/ml penicillin) in a 37°C, 5% CO_2_ environment. The medium was charged every 2 days. The second passage was used for subsequent experiments.

### RNA Isolation and Quantitative Real-Time RT-PCR

The NP tissue was washed briefly with physiologic salt solution thrice, and then frozen with liquid nitrogen. Total RNA was extracted from NP tissue and cell with Trizol reagent (Invitrogen, Carlsbad, CA) following the manufacturer, instructions. Quantification of RNA as based on spectrophotometric analysis at 260 nm wavelength. Ratios of absorbance at 260 and 280 nm of 1.9–2.0 confirmed the purity of the RNA samples. Reverse transcription was performed to synthesize cDNA using 2 µg of total RNA in a 20 µl reaction volume of M-MLV reverse transcriptional system (Invitrogen) in the presence of oligo(dT)_18_. The cDNA was used for quantitative real-time PCR (qrt-PCR) with specific gene primers in Table 1. SYBR green PCR mix (Transgen, Beijing, China) and iQ5 Real-Time PCR Detection System (Bio-Rad, CA) were used for the qrt-PCR. The real-time PCR system was composed of 1× SYBR Green I fluorescent dye (Takara, Dalian, China), 1 µl, upstream primers; 1 µl, downstream primers; 1 µl, 2× qPCR mix; 10 µl, sample cDNA 1 µl; and sterile double distilled water, 8 µl. The sequences of the specific primers are shown on Table 2. The reactions were incubated at 95°C for 30 s followed by 45 cycles of 95°C for 5 s, 60°C for 10 s, and 72°C for 30 s. The relative expression of the gene of interest was assessed by the ΔΔ*C*_t_ method.^16^ In this article GAPDH was chosen as an internal control.

**Table 1:** Clinical Finding in 45 Patients with LDH

Patient No.	Sex/Age (yr)	Type of LDH	Level	Duration of Symptoms (mo)	Classification	BMI
1	F/53	P	L5–S1	6	4	21.5
2	M/53	SE	L4–L5	72	5	25.7
3	M/26	S	L3–L4	26	2	21.0
4	F/42	TE	L4–L5	24	3	20.2
5	M/49	P	L5–S1	86	4	25.1
6	F/54	S	L4–L5	54	4	21.3
7	M/56	P	L4–L5	5	5	25.2
8	M/52	TE	L5–S1	2	4	23.4
9	M/49	TE	L4–L5	12	4	23.7
10	M/26	SE	L3–L4	2	3	25.0
11	F/53	SE	L4–L5	5	4	23.1
12	M/36	TE	L4–L5	1	2	23.5
13	F/46	P	L4–L5	48	3	23.1
14	F/51	S	L5–S1	86	4	22.9
15	F/41	S	L5–S1	84	3	21.8
16	F/51	SE	L4–L5	36	4	21.5
17	M/53	SE	L4–L5	60	5	30.3
18	F/55	SE	L3–L4	24	5	24.3
19	M/37	P	L4–L5	2	2	21.3
20	M/56	S	L4–L5	2	5	28.7
21	F/46	S	L4–L5	74	4	23.8
22	F/56	SE	L4–L5	48	5	28.5
23	M/42	P	L5–S1	12	2	22.3
24	M/34	P	L5–S1	37	2	21.2
25	M/42	P	L4–L5	120	4	26.9
26	M/44	S	L5–S1	72	4	25.2
27	M/56	SE	L3–L4	36	5	26.1
28	M/52	TE	L4–L5	36	4	26.9
29	M/52	P	L4–L5	1	4	25.2
30	F/56	TE	L3–L4	12	4	20.7
31	M/46	P	L5–S1	7	4	21.2
32	F/57	P	L5–S1	48	5	20.2
33	M/54	S	L4–L5	5	4	21.9
34	M/52	P	L4–L5	9	3	20.7
35	M/52	TE	L3–L4	11	4	21.4
36	F/38	S	L5–S1	3	3	28.1
37	F/55	P	L4–L5	48	4	21.9
38	F/32	S	L5–S1	6	3	19.3
39	F/39	S	L4–L5	61	4	19.5
40	M/48	SE	L4–L5	1	4	20.2
41	M/54	TE	L5–S1	6	4	19.4
42	F/48	SE	L5–S1	9	4	31.6
43	F/55	S	L4–L5	48	5	31.3
44	M/48	TE	L5–S1	8	4	30.2
45	M/55	TE	L5–S1	72	5	28.0

F, indicates female; LDH, lumbar disc herniation; M, male; P, protrusion; S, sequestration; SE, subligamentous extrusion; TE, transligamentous extrusion; L, lumbar; S, sacral; mo, months; yr, year, and BMI, body mass index.

**Table 2:** Nucleotide Sequences of Primers Used in Real-Time RT-PCR

Gene	Primer Sequence	*T*_m_ (°C)	Product Size (bp)
CA12	Forword: CGTGCTCCTGCTGGTGATCT	60	70
	Reverse: AGTCCACTTGGAACCGTTCACT		
OBRa	Forword: **TTGTGCCAGTAATTATTTCCTCTT**	56	200
	Reverse: **AGTTGGCACATTGGGTTCAT**		
OBRb	Forword: **CCAGAAACGTTTGAGCATCT**	56	609
	Reverse: **CAAAAGCACACCACTCTCTC**		
β-Actin	Forword: CTGGCACCACACCTTCTACA	56	110
	Reverse: AGCACAGCCTGGATAGCAAC		
Vimentin	Forword: GAAGAGGTTAGTGGAGTGA	56	131
	Reverse: TGCTGTTCCTGAATCTGA		
β-Tubulin	Forword: CACCTTGAATGGATGATGAACT	56	144
	Reverse:ATGAGGAAATAAACGAAGAAATGT		
GAPDH	Forword:TCAACGACCACTTTGTCAAGCTCAGCT	56	116
	Reverse: GGTGGTCCAGGGGTCTTAC		

### Western Blotting Analysis

To examine the change that may occur in NP cell due to biochemical stimulation, these cells were treated with 10 ng/ml leptin (Sigma–Aldrich, Oakville, ON, Canada), a concentration within the range of plasma concentrations found in obese individuals,^17^ for 24, 48, 72, and 96 h, respectively. Untreated cells were chosen as controls. Cell lysate were subject to SDS–PAGE and transferred to a PVDF membrane (Millipore, MA). Primary antibodies against the following proteins were used: leptin receptor (dilutions 1:1,000, Abcam, Cambridge, UK), β-actin (dilutions 1:2,000, Abcam), Vimentin (dilutions 1: 500, Bioworlde), β-tubulin (dilutions 1: 500, Bioworlde) and GAPDH (dilutions 1: 2,000, Abcam). HRP-conjugated secondary antibodies (dilutions 1: 1,000, Abcam) were used. Signal was detected using ECL kit (Millipore).

### Immunofluorescence Microscopy

Coverslips were placed into 24-well plate and then NP cells were plated and treated with 10 ng/ml Leptin for 48 h. After incubation and treatment, medium was removed and then cells were washed twice with PBS, fixed with 3.5% formaldehyde fixative for 30 min at 37°C. The cells were rinsed with PBS for 3 × 2 min, permeabilized with 0.1% Triton X-100 in PBS for 20 min and blocked with 3% BSA and 0.05% Tween 20 in PBS for 30 min at room temperature and then incubated overnight at 4°C with primary antibody. The antibodies used were as follows: type II collagen was stained with anti-type II collagen (dilutions 1: 250, Bioworlde); F-atin was stained with rhodamine–phalloidin (dilutions 1: 1000, Sigma–Aldrich); Vimentin was stained with anti-vimentin (dilutions 1: 250, Bioworlde); β-tubulin was stained with anti-β-tubulin (dilutions 1: 250, Bioworlde). The cells were treated with fluorescent anti-rabbit secondary antibody (dilutions 1:500, Bioworlde) for 2 h at room temperature. Nuclei were stained with 4, 6-diamidino-2-phenylindole (DAPI) (dilutions 1: 1,000, Sigma–Aldrich). Fluorescence images were acquired with a Leica TCS SP2 confocal microscopy (Leica, Mannheim, Germany) using the Leica Confocal Sofware.

### Statistical Analysis

Statistical analyses were performed using the SPSS 17.0 statistical software program. For human study, the Kruskal–Wallis test was used to assess the difference in the expression of OBRa and OBRb among disc specimens of different heriation types, and independent *t*-test to assess that between the specimens from different genders. The correlation between the expression of OBRa and OBRb and the age and BMI of the patients was determined by Pearson test, and that between the expression of OBRa and OBRb and duration of symptoms was determined by Spearman test. Data were expressed as means ± SD. Western blotting results were normalized with GAPDH. Independent experiments were performed twice. Statistical analysis was performed with Student's *t*-test. *P* values less than 0.05 were considered statistically significant.

## RESULTS

### Expression of Leptin Receptors in NP Tissues and Cells was Correlated with BMI

**Figure 1:**
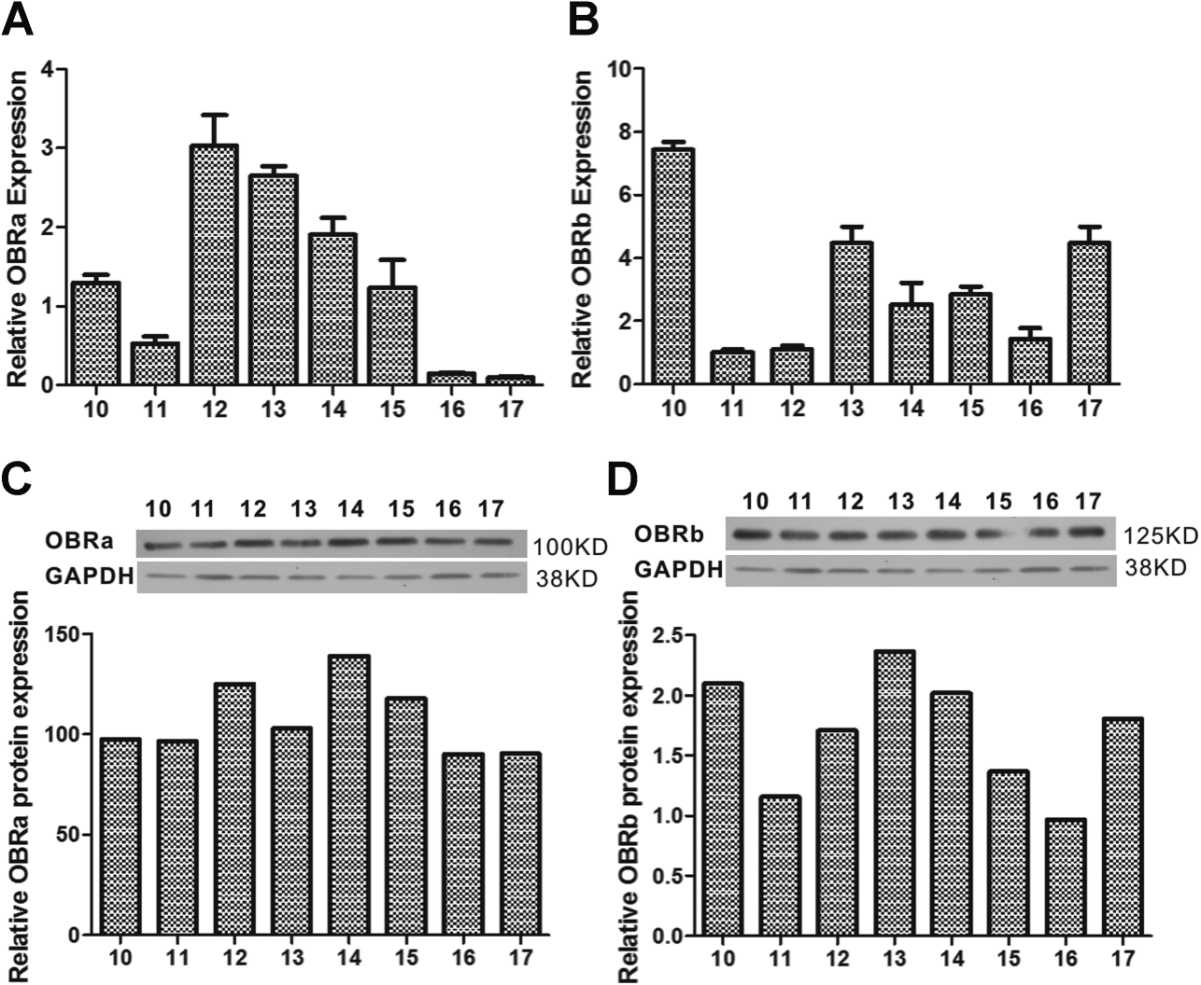
OBRa and OBRb expression in human NP cells. (A, B) Real-time RT-PCR analysis of OBRa and OBRb mRNAs in the human NP cells of eight patients. (C, D) Western blotting analysis of OBRa and OBRb protein expression in the human NP cells of eight patients. The signal in each lane was quantified using ImageJ software and the ratio of OBRs to GAPDH was determined.

**Figure 2:**
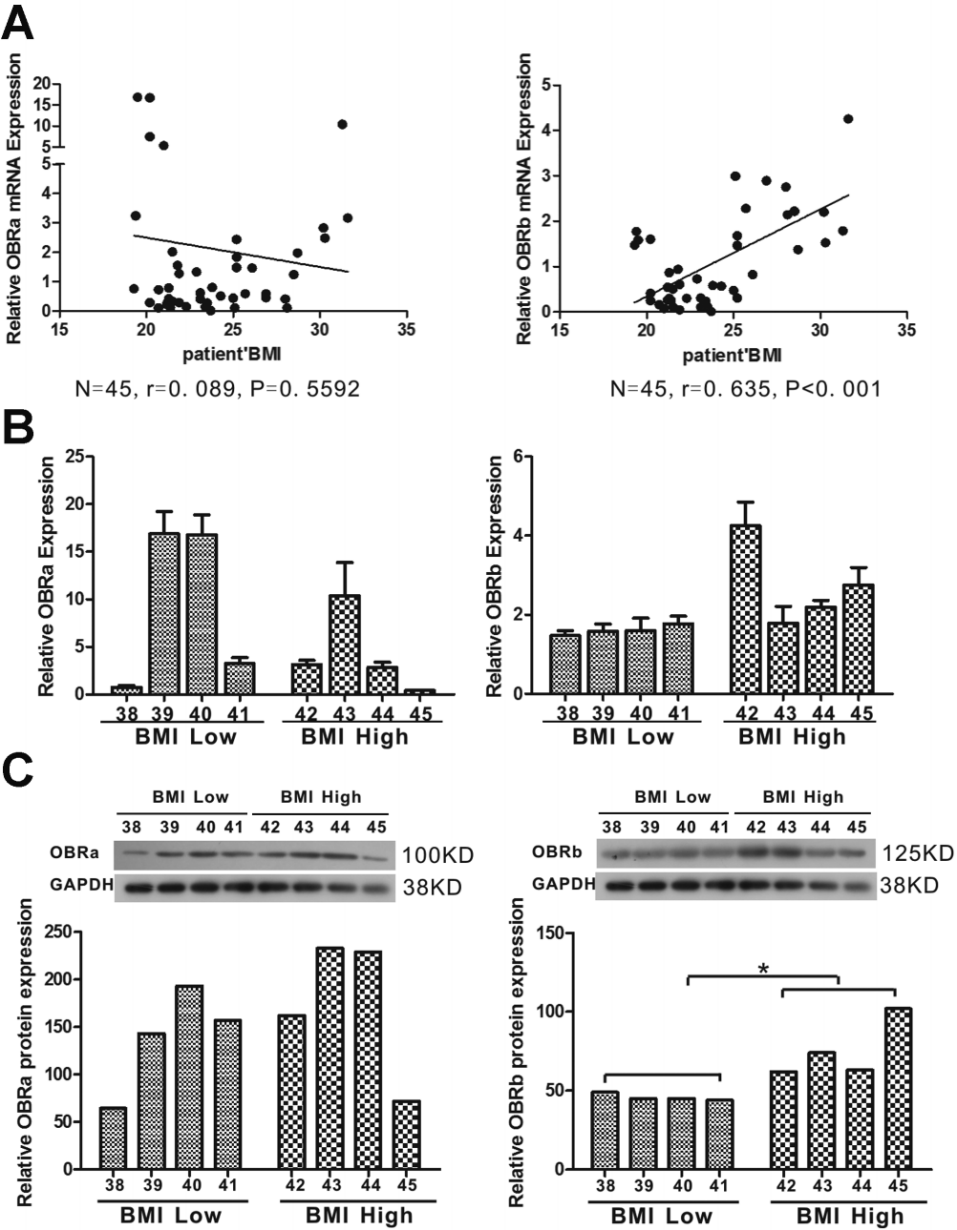
The correlation between the expression of OBRa and OBRb and patients' BMI. (A) The correlation between the expression of OBRa and OBRb and patients' BMI. (B) Real-time RT-PCR analysis of OBRa and OBRb mRNAs in the human NP tissue of low-BMI group (BMI = 19.3, 19.5, 22.6, and 22.2) and high-BMI group (BMI = 31.6, 31.3, 30.25, and 28.04). (C) Western blotting analysis of OBRs protein expression in the human NP tissue of low-BMI group (BMI = 19.3, 19.5, 22.6, 22.2) and high-BMI group (BMI = 31.6, 31.3, 30.25, 28.04). Data are present as mean ± SD (*n* = 3). Error bars represent SD. *p < 0.05, when the low-BMI group compared to high-BMI group.

The mRNA and protein of OBRa and OBRb were expressed in all human NP tissues and NP cells (Figs. 1 and 6). No significant differences were observed between samples from different herniation types or genders; the expression of OBRb but not OBRa was correlated with BMI of the patients (*r* = 0.635, *p* < 0.001; Fig. 2A); but not with the duration of symptoms or the age of the patients (Fig. 2A). The disc degeneration grade was not significantly correlated with BMI (*r* = 0.14, *p* = 0.45) or the expression of OBRa (*r* = 0.35, *p* = 0.36) and OBRb (*r* = 0.09, *p* = 0.09). For further validation, we determined OBRa and OBRb expression in an additional group of patients (*n* = 8) with different BMI. The patients were divided into two groups according to their BMI: high-BMI group (BMI = 31.6, 31.3, 30.25, and 28.04) and low-BMI group (BMI = 19.3, 19.5, 22.6, and 22.2). The mRNA expression and the protein expression of OBRa in the BMI-high group were significantly higher than that in the BMI-low group (*p* < 0.05) (Fig. 2B,C), but not the expression of OBRa. In additional, the expression level of OBRa and OBRb varied with different patients.

### Leptin Induced F-actin Formation and Increased β-actin Expression in NP Cells

**Figure 3:**
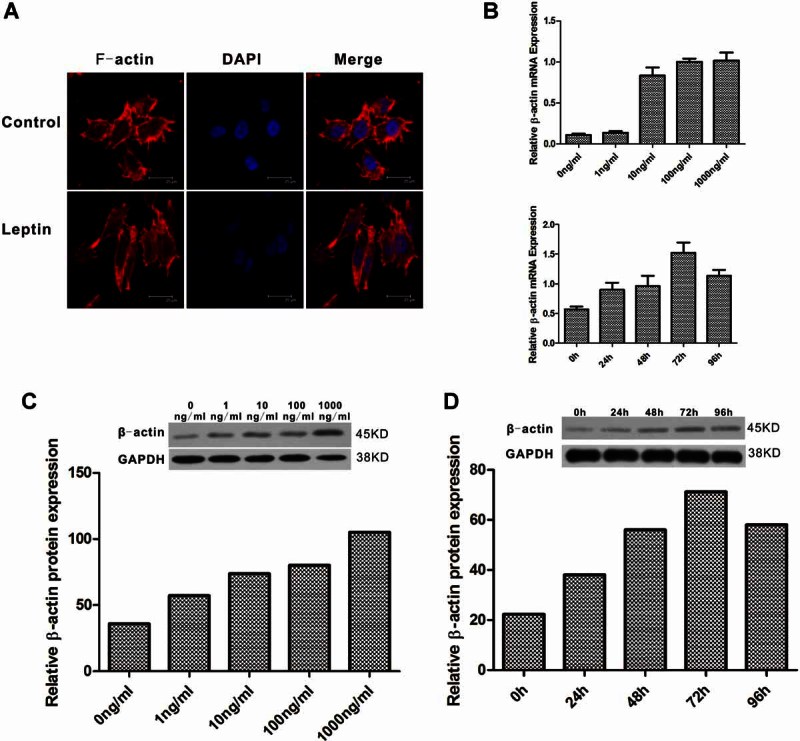
The effect of leptin on the F-actin cytoskeleton of human NP cells. (A) Fluorescence microscopy images showing arrangement of rhodamine–phalloidin-stained (red) F-actin filaments in primary human NP cells treated without or with leptin. Nuclei were stained with DAPI, shown in blue. Images were acquired using laser scanning confocal microscopy under a 40× objective. Control NP cells exhibited diffuses cytoplasmic and perinuclear staining of F-actin while leptin-stimulated NP cells showed strong cytoplasmic filamentous structures. Images are representative of three separate experiments. (B) The effect of leptin on β-actin mRNA expression assessed using real-time RT-PCR. For the dose-dependent studies NP cells were treated with either medium only or varying concentrations of leptin (1–1,000 ng/ml) for 24 h. For the time-dependent studies, NP cells were treated with either medium only or leptin (10 ng/ml) for varying time intervals (0–96 h). (C) The effect of leptin on β-actin protein expression assessed using Western blotting. For the dose-dependent studies quiescent NP cells were treated with either medium only or varying concentrations of leptin (1–1,000 ng/ml) for 24 h. For the time-dependent studies, quiescent NP cells were treated with either medium only or leptin (10 ng/ml) for varying time intervals (0–96 h).

In the untreated group, F-actin filaments of unstimulated NP cells were predominately localized beneath the cell membrane and exhibited a weak cytoplasmic and perinuclear staining. Interestingly, after the application of a 10 ng/ml leptin for 24 h, the cells had a significant change of cell shape. In this regard, the punctuate distribution of F-actin was lost, and replaced by a large member of F-actin stress fibers; F-actin in leptin-stimulated NP cells also showed more intense cytoplasmic staining with occasional localization along filamentous structures (Fig. 3A).

Treating NP cells with leptin demonstrated that leptin (10 ng/ml) significantly increased β-actin transcription in a time-dependent manner, with a maximal response at 72 h. Dose-dependent studies demonstrated a maximal response to leptin (1–1,000 ng/ml) was at the concentration of 1,000 ng/ml at the 24 h time point (Fig. 3B).

To further investigate whether the induction in β-actin mRNA was paralleled by an increase in protein level, Western blot was performed (Fig. 3C,D). Similar to the effect of leptin on β-actin mRNA, time- and dose-dependent effects of leptin on β-actin protein expression was observed.

### Leptin Increased Expression But Did Not Alter Organization of Vimentin in NP Cells

**Figure 4:**
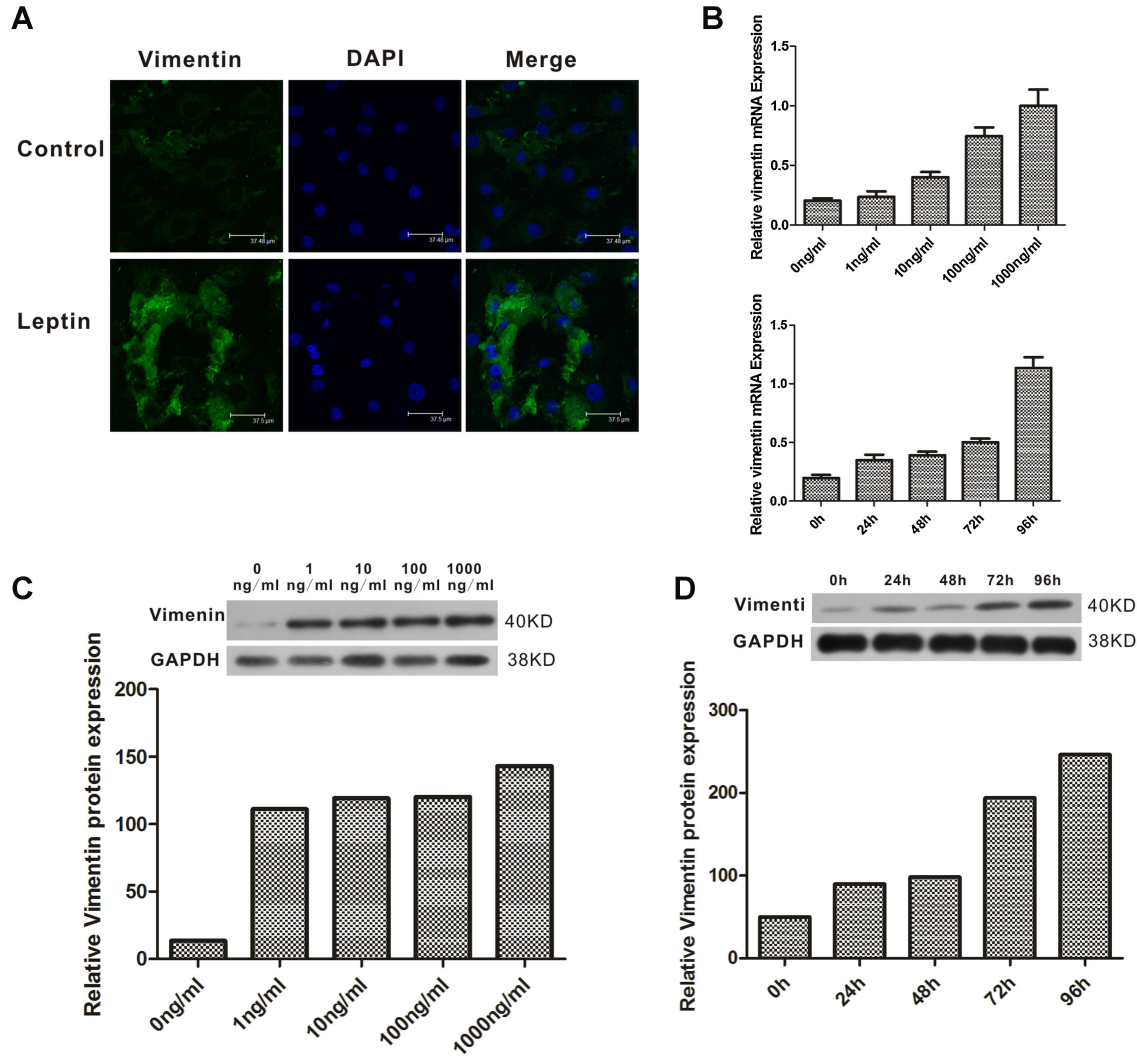
The effect of leptin on the vimentin cytoskeleton of human NP cells. (A) Fluorescence microscopy images showing arrangement of vimentin cytoskeleton (blue) in primary human NP cells treated without or with leptin. Nuclei were stained with DAPI, shown in blue. Leptin did not appear to alter vimentin filament organization, although slightly stronger labeling was observed in the leptin-induced group compared to the control group. (B) The effect of leptin on vimentin mRNA expression assessed using real-time RT-PCR. For the dose-dependent studies quiescent NP cells were treated with either medium only or varying concentrations of leptin (1–1,000 ng/ml) for 24 h. For the time-dependent studies, NP cells were treated with either medium only or leptin (10 ng/ml) for varying time intervals (0–96 h). (C) The effect of leptin on vimentin protein expression assessed using Western blotting. For the dose-dependent studies NP cells were treated with either medium only or varying concentrations of leptin (1–1,000 ng/ml) for 24 h. For the time-dependent studies, quiescent NP cells were treated with either medium only or leptin (10 ng/ml) for varying time intervals (0–96 h).

Vimentin filaments displayed a fibrous network radiating through the cytoplasm in NP cells. Leptin did not appear to alter vimentin filament organization, although slightly stronger labeling was observed in the leptin-induced group compared to the control group (Fig. 4A). Treatment with leptin (10 ng/ml) significantly increased vimentin mRNA level in a time-dependent manner, with a maximal response at 96 h. The maximal response to leptin (1–1,000 ng/ml) occurred at the concentration of 1,000 ng/ml at the 24 h time point (Fig. 4B). Time- and dose-dependent upregulation of vimentin proteins was also observed (Fig. 4C,D).

### Leptin Did Not Affect Expression or Organization of β-Tubulin in NP Cells

**Figure 5:**
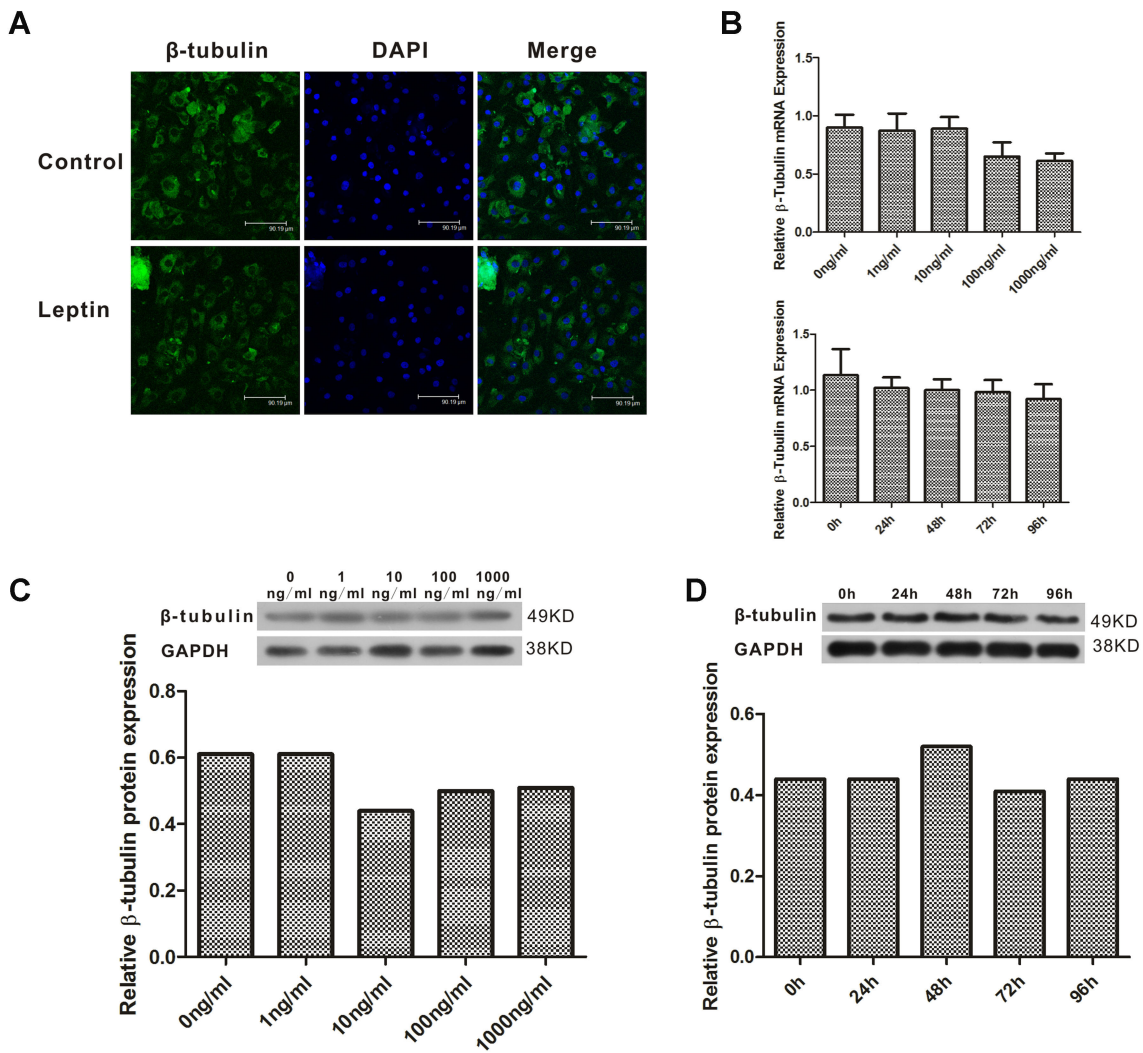
The effect of leptin on the β-tubulin cytoskeleton of human NP cells. (A) Fluorescence microscopy images showing arrangement of β-tubulin cytoskeleton (blue) in primary human NP cells treated without or with leptin. Nuclei were stained with DAPI, shown in blue. Images were acquired using laser scanning confocal microscopy under a 40× objective. Leptin did not appear to alter β-tubulin filament organization in the leptin-induced group compared to the control group. (B) The effect of leptin on β-tubulin mRNA expression assessed using real-time RT-PCR. For the dose-dependent studies quiescent NP cells were treated with either medium only or varying concentrations of leptin (1–1,000 ng/ml) for 24 h. For the time-dependent studies, quiescent NP cells were treated with either medium only or leptin (10 ng/ml) for varying time intervals (0–96 h). (C) The effect of leptin on β-tubulin protein expression assessed using Western blotting. For the dose-dependent studies quiescent NP cells were treated with either medium only or varying concentrations of leptin (1–1,000 ng/ml) for 24 h. For the time-dependent studies, quiescent NP cells were treated with either medium only or leptin (10 ng/ml) for varying time intervals (0–96 h).

The architecture of the β-tubulin networks, as well as mRNA and protein expression of β-tubulin, was determined in the NP cells treated with or without leptin. Analysis of the confocal images indicated that there was no obvious difference in β-tubulin organization between the two groups (Fig. 5A). The β-tubulin mRNA expression and protein level was unaffected at all time points and all concentration points analyzed (Fig. 5B–D).

## DISCUSSION

**Figure 6:**
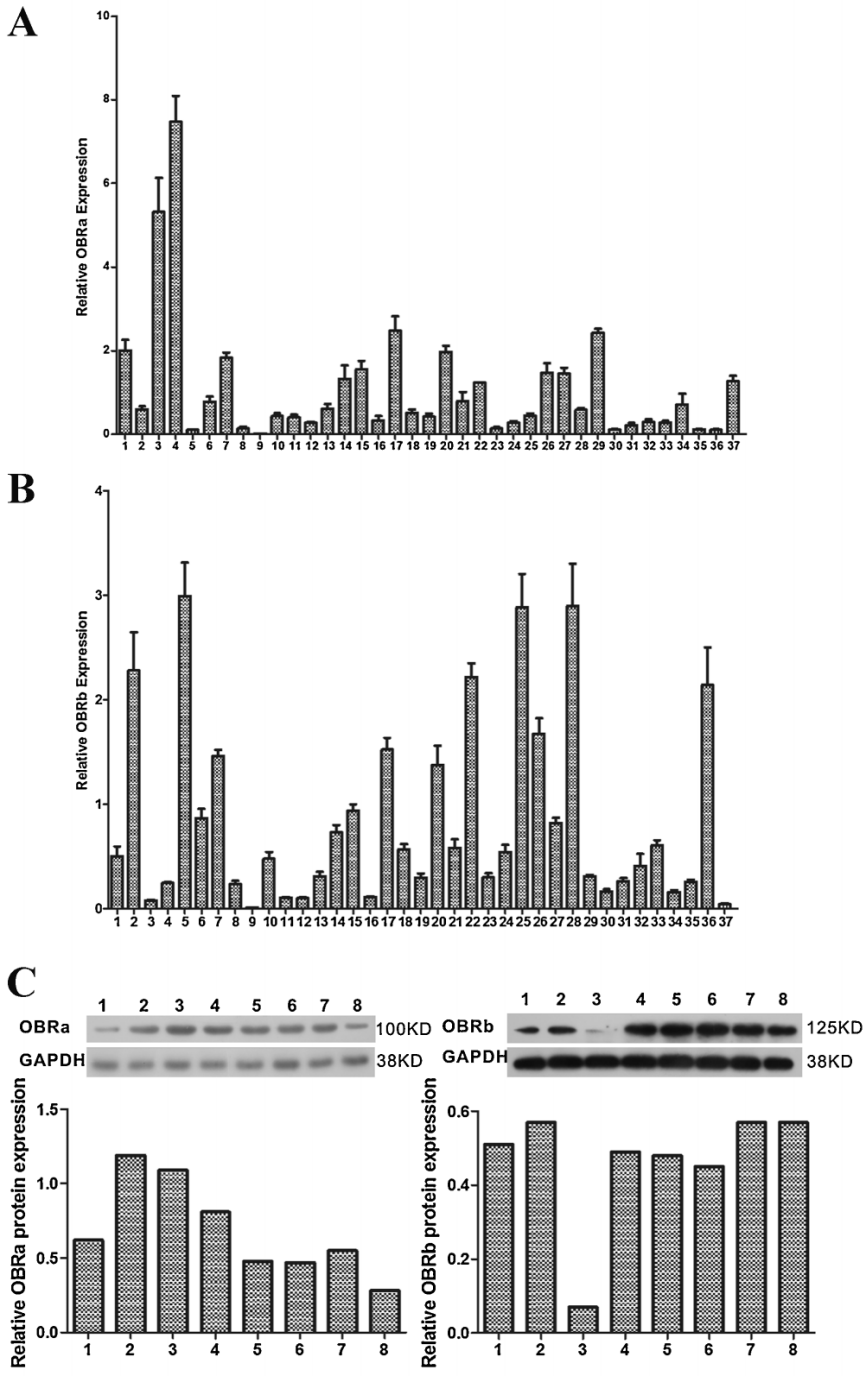
OBRa and OBRb expression in human NP tissue. Real-time RT-PCR analysis of OBRa (A) and OBRb (B) mRNAs in the human NP tissue of 37 patients. Real-time RT-PCR analysis was performed in triplicate and the expression levels of OBRa OBRb mRNAs were normalized to GAPDH mRNAs. Error bars represent standard derivation. (C) Western blotting analysis of OBRa and OBRb protein expression in the human NP tissue of eight patients. The signal in each lane was quantified using Image software and the ratio of OBRa and OBRb to GAPDH was determined.

Accumulating evidence from epidemiological studies indicates that high body mass index (BMI) increases the risk of lumbar disc degeneration (LDD).^18^ The mechanism by which overweight causes LDD is poorly understood. The contribution of both mechanical and systemic factors has been suggested. On the one hand, overweight directly increases the mechanical load on the spine. On the other hand, obese people have elevated serum levels of leptin, a known marker of inflammation and closely associated with cardiovascular, cancer and osteoarthritis risks.^19^ The role of hyperleptinemia as a contributing factor for increased risk of LDD in obesity, however, is not well understood. It has been reported that human intervertebral disc cells can secret detectable level of leptin and disc cells express functional leptin receptor.^9^, ^10^ The results of our study demonstrate for the first time that disc tissues and NP cells express OBRa and OBRa. The expression of OBRb in disc tissues is also dependent on body weight. In vitro studies suggest that leptin alters the organization and induces mRNA and protein levels of the cytoskeletal elements in NP cells (Fig. 6).

OBR is a member of the class I cytokine receptor super family, receptors of which lack intrinsic tyrosine kinase activity and are activated by ligand-induced receptor homo- or hetero-oligomerization. Leptin acts through its receptor, the product of the db gene, which has at least six isoforms.^20^ Mutation in OBR causes early onset obesity, hyperphagia and hypothalamia.^21^ In comparison with other five short isoforms of leptin receptors, the role of long signaling form (OBRb) in leptin signaling is well studied and recognized as the main signal mediator of leptin. The almost universal distribution of OBRa and OBRb reflects the multiple biological effects in nonadipose tissues, providing evidence for the extensive functional pleiotropy of leptin. To investigate the two major leptin receptor isoforms (i.e., OBRa and OBRb) in the development of LDD, we quantified OBRa and OBRb mRNA expression using an accurate and sensitive real-time RT-PCR method. We also quantified OBRs protein expression. Our study revealed the presence of OBRa and OBRb mRNA and protein expression in all patients' NP cells. The levels of OBRa and OBRb mRNA and protein expression were found to be similar between different herniation types, genders, levels and duration of symptoms, which is consistent with the previous reports.^10^ In this respect, previous studies had shown that disc cells express leptin functional receptor and the total percentage of leptin receptor-positive cells is correlated with the age of the patients. To our knowledge, the existing reports only used immunostaining methods for the detection of the leptin functional receptor in human NP tissue. We quantified, for the first time, OBRa and OBRb mRNA expression using an accurate and sensitive real-time RT-PCR method. Our results suggested that lumbar NP tissues and NP cells are capable of receiving leptin signals, at least in part, via these two OBRs. Based on our real-time RT-PCR and Western blot results, OBRa and OBRb mRNA and protein expression in NP tissue and NP cells are commonly expressed, suggesting that the in vitro data may be extended to the in vivo situation. In addition, we demonstrate a considerable increase of OBRb mRNA and protein expression with body weight. This is consistent with existing literature showing that the expression of OBRb mRNA in liver is dependent on the body weight.^22^ Previous study also demonstrated that cultured astrocytes expressed mRNA for the leptin receptor, which increased in response to leptin treatment.^23^ Thus, the higher level of expression of OBRb in the NP tissue seems to be related to hyperleptinemia in obesity. However, the disc degeneration grade was not correlated significantly with BMI and the expression of OBRa and OBRb in these operation patients. Moreover, the expression level of OBRb varied greatly among patients and the mRNA expression of OBRb is substantially reduced in some high BMI patients. Furthermore, it has been reported that leptin resistance occurs in severely obese humans and the reduction of circulating OBRs in obese subjects may contributes to the leptin resistance.

After confirming the expression of leptin and its receptors in NP cells, we investigated the effect of leptin on the organization and expression of three major cytoskeleton elements in NP cells in vitro. Our data demonstrated that leptin induced F-actin rearrangement. A great number of actin stress fibers were visible in NP cells, which are comparable to a previous study on chondrocytes.^13^ Leptin increased β-actin mRNA levels. The induction was also evident at the protein level in NP cells as assessed by Western blot. These results in this study are similar to those reported by other investigators: (1) Liang et al.^13^ demonstrated that leptin activated the RhoA/ROCK/LIMK/confilin pathway and induced cytoskeleton reorganization in chondrocytes; (2) Li et al.^24^ demonstrated that tensile strain remodeled the F-actin cytoskeleton in NP cells. Actin microfilaments are involved in many cellular processes including alteration of cell shape, movement of organelles, cell migration and adhesion, endocytosis, secretion, contractile ring formation, and extracellular matrix assembly.^25^ Furthermore, it has been demonstrated that cytoskeleton may play a crucial role in mechnotransduction between the IVD cells and their surrounding extracellular matrix. More importantly, microfilaments provide the viscoelastic properties of the chondrocyte, and changes in the structures and properties of these cytoskeleton elements may reflect changes in the chondrocyte with osteoarthritis.^26^ β-Actin had altered architectures and expression levels in NP cells from young and skeletally mature IVD.^12^ Together, these findings suggest that leptin induces F-actin remodeling and increase mRNA and protein expression of β-actin and may act as a mediator for transducting mechanical signals and has a potential role in intervertebral disc degeneration.

In our study, vimentin organization did not alter appreciably in response to leptin in NP cells, although there was evidence of increased vimentin staining in cell processes. Indeed, leptin also increased vimentin mRNA expression and this alteration was also reflected at the protein level. Similar effect of leptin has been observed in hypothalamic astrocytes.^27^ The main functions of vimentin filaments are to maintain cell shape and its elastic properties in resisting mechanical forces. Vimentin, intermediate in size between the microfilaments and microtubules, forms highly organized fibrous protein structure, which appears to connect the cell periphery with the nucleus.^28^ Several lines of evidence suggest that alterations in vimentin structure correlate with the progression of intervertebral disc degeneration. For example, a preliminary study had shown that the majority of these vimentin–immunopositive cells were appeared to emanate short cytoplasmic processes in discs from patients with low back pain or scoliosis compared to discs from normal.^29^ Normal versus osteoarthritic cartilages shown a reduction in the percentage of labeled chondrocytes of 37.1% for vimentin.^30^ Disruption of vimentin reduced stiffness approximately 2.8-fold in normal chondrocytes. It has been reported that AF cells responded to static compression with increased expression of vimentin mRNA as well as increased polymerization of vimentin subunits.^31^ Hence, leptin-induced expression of vimentin observed in our study suggests that vimentin plays an important role in the cellular reponse to leptin. In contrast to the observed remodeling of F-actin, the β-tubulin architecture in NP cells was unresponsive to leptin. Very few studies have reported the variations in β-tubulin cytoskeleton in the intervertebral disc, but one report indicated that there was no age-related difference in the expression of β-tubulin mRNA and protein in the NP cells.^12^ More recently, another report suggested that strain did not appear to alter β-tubulin mRNA levels and protein synthesis in NP cells.^12^, ^24^

A variety of biochemical mediators of inflammation and tissue degeneration, including matrix metalloproteinases, prostaglandin E_2_, nitric oxide, and a variety of cytokines such as interleukin-1 and interleukin-6 were found to be involved in the matrix breakdown of articular cartilage.^32^ Our findings suggest that leptin may play an important role in disc degeneration. For instance, it has been shown that leptin is secreted by the adult intervertebral disc, which is avascular. Our finding that lumbar NP tissues and NP cells produce OBRa and OBRa is very exciting because it suggests the presence of a local autocrine or paracrine mechanism within the disc tissues. More importantly, our result suggest that the increased expression of OBRb in lumbar NP tissues and cells in obesity may participate in leptin transport within the intervertebral disc itself and it has been increased in response to leptin treatment. Alteration in cytoskeletal remodeling has been implicated in the pathogenesis of LDD. For example, static compression and cyclic strain, two important physical stimuli exposed to IVD cells, have been found to modulate cytoskeletal remodeling in intervertebral disc cells.^24^, ^33^ Change of cytoskeleton may in turn alter the expression of other extracellular matrix (ECM) proteins such as type I collagen, type II collagen and aggrecan^34^. These findings suggest that this is a direct linkage between cytoskeletal remodeling in NP cells and the disease development of LDD. In the present study, increased stress fiber formation and vimentin filaments are observed in leptin-treated NP cells. It has been suggested that the phenotypic stability of NP cells is critical and that modifications to NP cells may modulate intracellular signaling pathways upstream of ECM gene transcription. It is therefore, interesting to examine if blockade of leptin function (e.g., by inhibitors to OBRs) will modify the effect of obesity on LDD development in vivo. One of the drawbacks of this study is the lack of age-matched non-degenerate discs as control. Second, NP cells derived from LDD patients may not necessarily reflect the in vivo scenario. Third, this study only evaluated the effect of leptin on LDD NP cells. The comparison of the reaction of LDD and normal NP cells to leptin stimulation should provide further information for the potential involvement of leptin in LDD development.

In conclusion, our results show for the first time that lumbar NP tissues and NP cells produce OBRa and OBRa and an increase in body weight generally causes an increase in the OBRb mRNA expression in NP cells. Further, we have demonstrated that leptin induces cytoskeletal reorganization and upregulates mRNA and protein levels of cytoskeletal elements in NP cells. These findings provide evidence supporting the possible involvement of leptin in the pathogenesis of LDD. It is anticipated that, with a further understanding of the complex biology of LDD and its relationship with obesity, functional inhibition of the leptin signal may be a hopeful strategy for the prevention and treatment of LDD.

## Supplementary material

Supplemental Figure 1. Pfirrmann classification of lumbar disc degeneration. (A)Grade II: The structure of the disc is inhomogeneous, with a hyperintense white signal. The distinction between nucleus and anulus is clear, and the disc height is normal, with or without horizontal gray bands. (B)Grade III: The structure of the disc is inhomogeneous, with an intermediate gray signal intensity. The distinction between nucleus and anulus is unclear, and the disc height is normal or slightly decreased. (C)Grade IV: The structure of the disc is inhomogeneous, with an hypointense dark gray signal intensity. The distinction between nucleus and anulus is lost, and the disc height is normal or moderately decreased. (D)Grade V: The structure of the disc is inhomogeneous, with a hypointense black signal intensity. The distinction between nucleus and anulus is lost, and the disc space is collapsed.
