# Supplementary figures and images for: The Role of Leptin on the Organization and Expression of Cytoskeleton Elements in Nucleus Pulposus Cells
Source: J Orthop Res. 2013 Jan 17;31(6):847–57. doi: 10.1002/jor.22308 (PMC3664408; doi:10.1002/jor.22308)

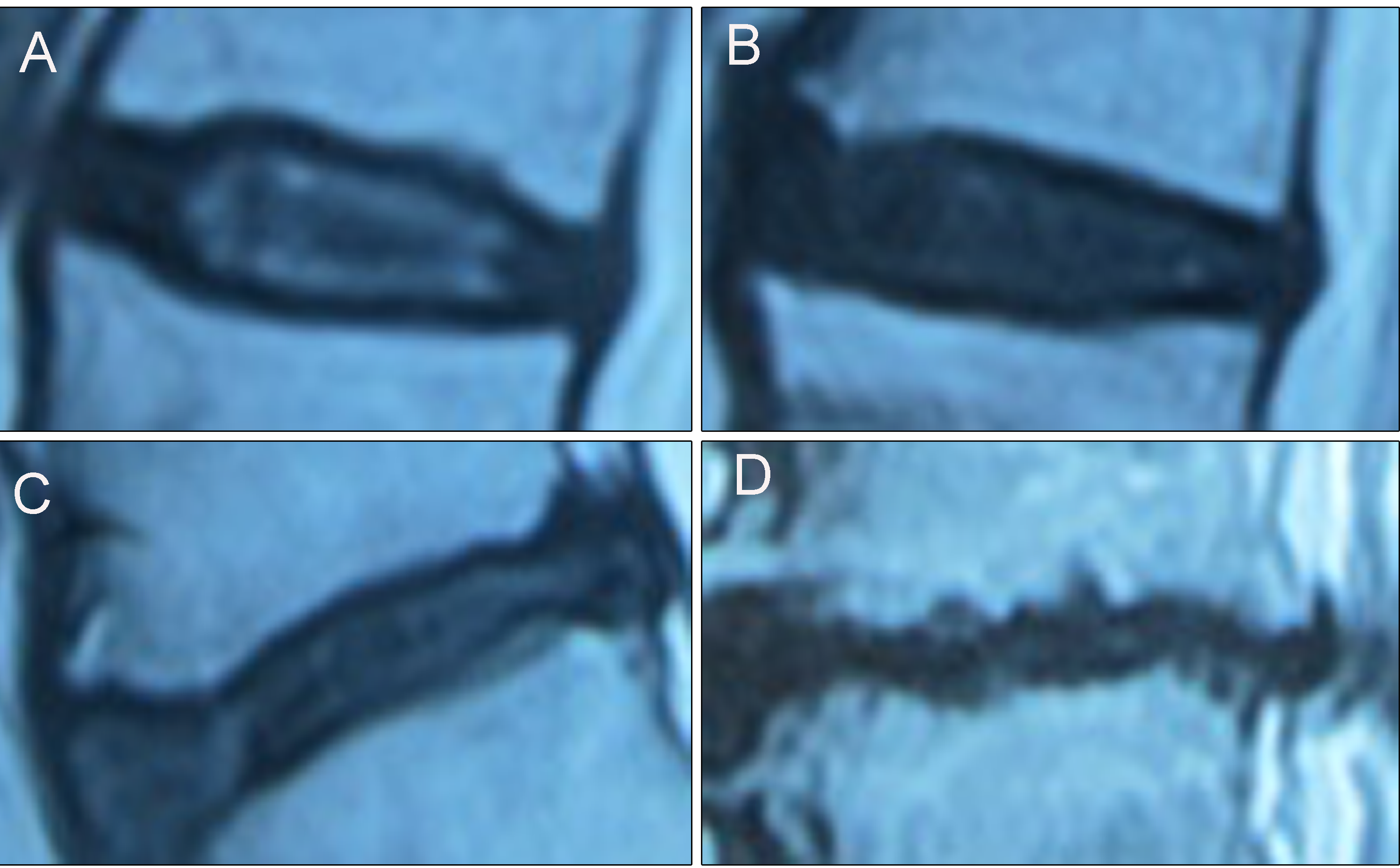

Supplement: Supplementary file 1 [file jor0031-0847-SD1.tif]
